# Design, Synthesis, Biological Activities and 3D-QSAR of New *N,N'*-Diacylhydrazines Containing 2,4-Dichlorophenoxy Moieties

**DOI:** 10.3390/molecules181214876

**Published:** 2013-12-03

**Authors:** Guo-Xiang Sun, Zhao-Hui Sun, Ming-Yan Yang, Xing-Hai Liu, Yi Ma, Yun-Yang Wei

**Affiliations:** 1 School of Chemical and Biological Engineering, Yancheng Institute of Technology, Yancheng 224051, Jiangsu, China; E-Mail: sunguoxiangyit@gmail.com; 2 School of Chemical Engineering, Nanjing University of Science and Technology, Nanjing 210094, Jiangsu, China; E-Mail: ywei@mail.njust.edu.cn; 3 College of Chemical Engineering and Materials Science, Zhejiang University of Technology, Hangzhou 310014, Zhejiang, China; E-Mails: esunzhaohui@126.com (Z.-H.S.); yangmingyanzjut@126.com (M.-Y.Y.); 4 State-Key Laboratory of Elemento-Organic Chemistry, National Pesticidal Engineering Centre (Tianjin), Nankai University, Tianjin 300071, China; E-Mail: mayink@126.com

**Keywords:** *N,N'*-diacylhydrazines, herbicidal activity, plant growth regulate activity, synthesis, 3D-QSAR

## Abstract

A series of new *N,N'*-diacylhydrazine derivatives were designed and synthesized. Their structures were verified by ^1^H-NMR, MS and elemental analysis. The herbicidal activities and plant growth regulating activity of these *N,N'*-diacylhydrazines were evaluated. The herbicidal activity results showed that most of these *N,N'*-diacyl-hydrazines showed excellent *in vivo* activities against *Echinochloa crus-galli*, *Digitaria sanguinalis*, *Brassica napus*, *Amaranthus retroflerus*. Most of them exhibited higher herbicidal activities against dicotyledonous weeds than monocotyledonous weeds. To further investigate the structure-activity relationship, comparative molecular field analysis (CoMFA) was performed on the basis of herbicidal activity data. Both the steric and electronic field distributions of CoMFA are in good agreement in this work.

## 1. Introduction

**Figure 1:**
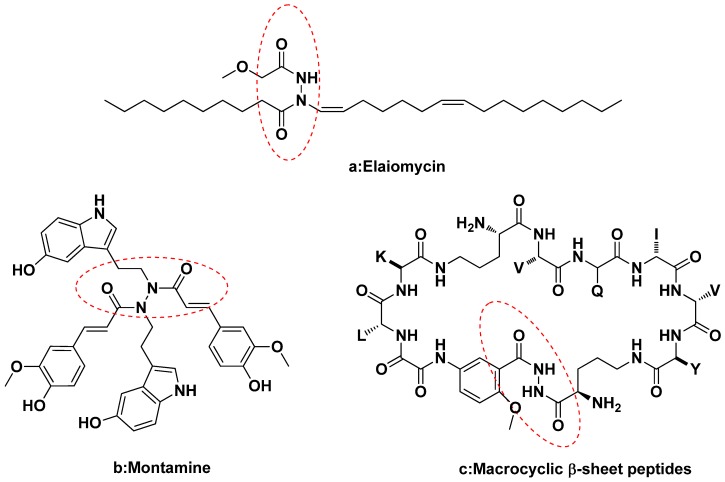
The natural products containing acylhydrazine structures.

In recent years, acylhydrazine derivatives have become one of the focuses in the development of agrochemicals due to their high biological activities [1,2,3,4,5], especially insecticidal activities. For example, *N*-*tert*-butyl-*N,N'*-diacylhydrazines are a new class of insect growth regulators that have been found to mimic the action of 20-hydroxyecdysone to activate the ecdysone receptor, leading to lethal premature moulting [6,7,8]. *N,N'*-Diacylhydrazines also display other activities, such as fungicidal activity [9], herbicidal activity [10], anti-HIV [11], anti-tumor activity [12] and so on. Many natural products contain diacylhydrazine groups. Some natural product examples of diacylhydrazines are presented in Figure 1. For example, Elaiomycin (a) is isolated from submerged culture filtrates of *Streptomyces gelaticus*; it displays strong *in vitro* inhibition of virulent and avirulent forms of the bovine and human strains of *Mycobacterium tuberculosis* [13]. Montamine (b), isolated from *C. montana*, and it exhibits anti-oxidation activity [14]. Macrocyclic β-sheet peptides (c) inhibit the aggregation of a tau-protein-derived hexapeptide [15].

The phenoxyacetic acids have widely used as herbicides in agriculture for over 30 years. They include 2,4-dichlorophenoxyacetic acid (2,4-D), 2-methyl-4-chlorophenxyacetic acid (MCPA), 4-chlorophenoxyacetic acid (4-CPA), *etc*. The synthesis of modified phenoxyacetic acid derivatives is a research hotspot [16,17,18]. It is reported that 2,4-D derivatives exhibit diversity biological activities. For example, 1-(substituted phenoxyacetoxy)alkylphosphonates display excellent herbicidal activity [19]. Furthermore, some substituted aryloxyacetic acid derivatives also exhibit good plant growth-regulating activity [20], brassinosteroid biosynthesis inhibitor activity [21], antifungal activity [22,23], herbicidal activities [24], and are affordable antitubercular agents [25].

**Figure 2:**
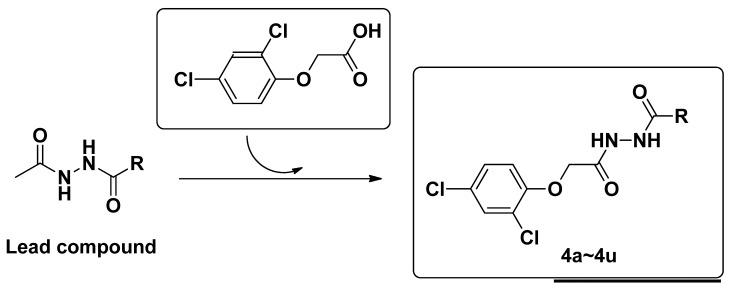
Design strategy of title compounds.

In view of these facts mentioned above, and also as a part of our continuing work [26] on the synthesis of bioactive lead compounds, the title compounds were designed by introducing the 2,4-dichlorophenoxy acetic acid pharmacophore into a diacylhydrazine scaffold. Our original strategy is depicted in Figure 2. Twenty-three novel diacylhydrazine derivatives were synthesized and characterized by ^1^H-NMR, MS and elemental analysis. The herbicidal activity and plant growth regulate activity of the diacylhydrazine compounds were tested *in vivo*.

## 2. Results and Discussion

### 2.1. Synthesis and Characterization

**Scheme 1:**
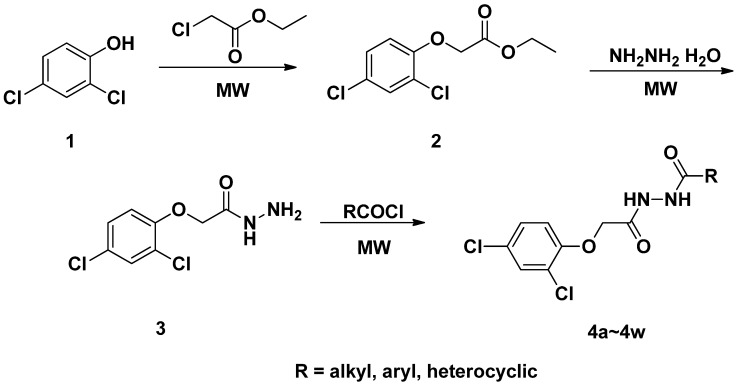
Synthetic route to the title compounds.

The synthetic route of the title compounds is outlined in Scheme 1. First, the title compounds were synthesized under conventional conditions. 2,4-Dichlorophenol, K_2_CO_3_, DMF and ethyl 2-chloro-acetate were placed in a round-bottomed flask and the mixture was stirred overnight at room temperature. When the reaction was complete, the mixture was poured into water, and compound **2** was collected. Next, to a solution of compound **2** in ethanol excess 80% hydrazine hydrate was added. The mixture was refluxed for 5 h, cooled to room temperature and the precipitate formed was filtered off to afford the pure product **3**. For the last step 3, Et_3_N and a substituted acyl chloride were mixed in THF and refluxed for 4 h to afford the crude solid. The target compounds were recrystallised from ethanol to afford the pure products. In order to optimize the reaction condition and reaction times, microwave irradiation was employed next. In the synthesis process of compound 2, the microwave method is used KI as catalysis, in order to accelerate reaction. The key intermediates **2**, **3** can be obtained in this way in excellent yield (>95%) after short reaction times (Table 1).

In the ^1^H-NMR spectra of title compounds, the CH_2_ proton signals sppeared at *δ* 4.57~4.82 ppm. The NH proton was observed as two single peaks, although sometimes, it appeared as one broad peak. All the other alkyl or aryl groups showed the normal peak locations. All the mass spectra of the title compounds show the molecular ion peaks.

**Table 1:** Comparison of yields of intermediates through methods with or without microwave irradiation.

No.	Method	Time	condition	Yield/%
**2**	No-MW	24 h	r.t.	88
	MW	4 min	200 W	95
**3**	No-MW	5 h	reflux	90
	MW	1 min	500 W	96

### 2.2. Herbicidal Activities

The herbicidal activities of these compounds were determined *in vivo*. The results for compounds **4a**–**x** are summarized in Table 2. As shown in Table 2, all the compounds exhibit excellent post-emergence herbicidal activity against *Amaranthus retroflexus* and *Brassica napus*, except **4r**. Most of these compounds alsoshow better herbicidal activities in post-emergence treatment than in pre-emergence treatment against *Amaranthus retroflexus* and *Brassica napus*. On the other hand, these compounds exhibited low activity against *Echinochloa crus-galli*, *Digitaria sanguinalis* under post-emergence conditions, but in pre-emergence conditions, **4p**, **4q**, **4r**, **4u**, **4v**, **4w** exhibited good herbicidal activity against *Echinochloa crus-galli*. Notably, alkyl substituted compounds or pyridine substituted compounds show surprisingly higher pre-emergence herbicidal activity against the monocotyledon *Echinochloa crus-galli* than the corresponding aryl substituted compounds. At 1,500 g ai/ha, the compounds **4a**–**x** exhibited the same herbicidal activities as seen in our previous work [26].

The subsequent results in Table 3 showed that the compound **4a** had good herbicidal activity against *Echinochloa crus-galli*, *Digitaria sanguinalis*, *Brassica napus*, *Amaranthusretroflerus* at a dosage of 750 g ai/ha. The herbicidal activities against dicotyledon weeds are comparable with the commercial herbicide 2,4-D. Surprisingly, cmpound **4a** exhibited good activities against monocotyledon weeds, so a further bioassay was performed. At dosage of 350 g ai/ha, it can be found that the title compound has low effective inhibition against all tested monocotyledon weeds. The compound **4a** displayed higher activities than that seen in the previous work for (*N'*-(2-(2,4-dichlorophenoxy)propanoyl)cyclopropanecarbohydrazide) [26] at the dosage of 350 g ai/ha. It can be found that the title compound **4a** has the most effective inhibitory activity against all tested dicotyledon weeds, even at a dosage as low as 187.5 g ai/ha. Under pre-emergence conditions, compound **4a** exhibited better activity against *Amaranthus* *retroflerus* and *Brassica napus* than that of the control 2,4-D.

**Table 2:** The herbicidal ^c^ and plant growth regulatory ^c^ activity of title compounds.

No.	R	CRC	*Ech.*		*Bra.*		*Dig.*		*Ama.*	
			Pre-	Post-	Pre-	Post-	Pre-	Post-	Pre-	Post-
**4a**	cyclopropyl	−100	6.2	15.0	92.9	100	72.5	30.0	100	100
**4b**	phenyl	−100	5.4	26.3	86.9	100	52.9	20.0	100	100
**4c**	*p*-nitrophenyl	−100	0	10.0	71.7	100	21.6	0	100	100
**4d**	*p*-chlorophenyl	−100	19.5	18.9	11.2	100	53.7	10.7	91.6	100
**4e**	*p*-fluorophenyl	−100	0	28.5	12.4	100	100	0	95.8	100
**4f**	*m*-methylphenyl	−100	12.1	27.3	32.0	100	11.1	7.1	91.6	100
**4g**	*m*-chlorophenyl	−100	0	6.5	33.7	100	16.7	21.4	87.4	100
**4h**	*o*-fluorophenyl	−100	11.6	19.4	100	100	37.0	21.5	89.5	100
**4i**	*o*-chlorophenyl	−100	9.1	22.3	64.5	100	64.8	7.1	91.6	100
**4j**	2,4-dichlorophenyl	−100	21.0	0	56.8	100	50.0	0	78.9	100
**4k**	*o*-methoxyphenyl	−100	0	21.7	49.1	100	22.2	3.6	81.1	100
**4l**	*p*-methoxyphenyl	−100	16.6	20.0	62.1	100	48.1	21.4	100	100
**4m**	*p*-iodophenyl	−100	23.5	0	52.1	100	3.7	3.6	70.5	100
**4n**	5-methylisoxazole-4-yl	−100	8.1	9.1	100	100	38.9	0	78.9	100
**4o**	1-CN-cyclopropyl	−100	20.5	14.9	14.8	100	0	17.9	49.5	100
**4p**	propyl	−100	100	27.9	100	100	87.0	28.6	100	100
**4q**	isopropyl	−100	100	20.0	100	100	70.4	21.4	83.2	100
**4r**	2,4-dichlorophenoxymethyl	−63.5	100	7.6	18.3	58.6	25.9	0	78.9	100
**4s**	(2-(2,4-dichlorophenoxy)-acetyl)propyl	−100	5.6	0	56.2	100	87.0	3.6	87.4	100
**4t**	furan	−100	0	7.6	100	100	63.0	17.9	85.3	100
**4u**	(2 *E*,4*Z*)-hexa-2,4-diene-	−100	100	31.8	100	100	31.5	0	93.7	100
**4v**	3-pyridine	−100	100	20.0	100	100	25.9	0	100	100
**4w**	4-pyridine	−100	100	17.2	100	100	66.7	10.7	83.2	100
**4x**	Methyl	−100	68.2	26.2	88.8	100	87.0	0	70.5	100
2,4-D		65.7	100	85.4	100	100	100	100	81.1	100

^a^ Ech: *Echinochloa crus-galli*; Bra: *Brassica napus*; Dig: *Digitaria sanguinalis*; Ama: *Amaranthus retroflerus*; ^b^ Pre: pre-emergence; Post: post-emergence; ^c^ The test concentration of herbicidal activity is at 1,500 g ai/ha, and the cotyledon root of cucumber (CRC) is at 10 mg/mL.

**Table 3:** Herbicidal activities of compound **4a** and 2,4-D (percent inhibition, %).

No.	Rate g/ha	*Ech.*		*Bra.*		*Dig.*		*Ama.*	
		Pre	Post	Pre	Post	Pre	Post	Pre	Post
**4a**	187.5	0	37.7	55.7	100	0	8.3	100	100
	375	0	50.4	53.3	100	0	12.7	100	100
	750	0	61.2	59.1	100	89.0	31.5	100	100
**2,4-D**	187.5	0	0	0	100	0	0	0	100
	375	0	0	0	100	0	0	52.4	100
	750	32.6	32.4	0	100	50.0	0	78.6	100

### 2.3. Plant Growth Regulatory Activity

In order to elucidate why these compounds had excellent herbicidal activity, the activity of title compounds on cotyledon root of cucumber was determined. Surprisingly, as listed in Table 2, all the tested compounds showed 100% inhibition on the root cotyledon of cucumber. Such strong inhibition of plant root growth can possibly the reason behind the aforementioned excellent herbicidal activity.

### 2.4. CoMFA Analysis

The CoMFA method is widely used in drug design, because it allows for rapid prediction of QSAR of newly designed molecules [27]. The results of these computations are summarized in Table 4.

**Table 4:** Summary of CoMFA analysis.

method	q^2^	r^2^	S	F	No.	Contributor (%)	
						Steric	Electrostatic
CoMFA	0.57	0.886	0.435	97.628	**4f**	66.9%	33.1%

**Figure 3:**
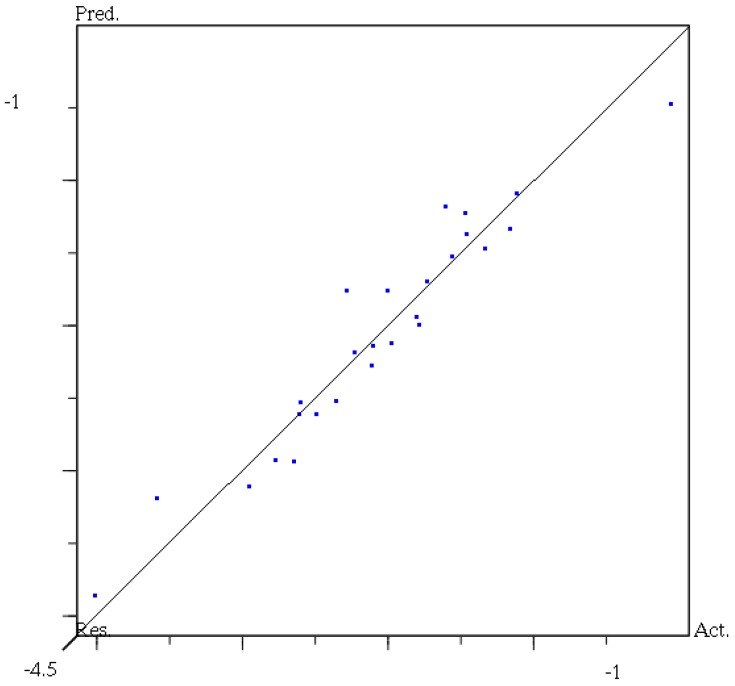
CoMFA predicted as experimental *p*IC_50_ values.

**Figure 4:**
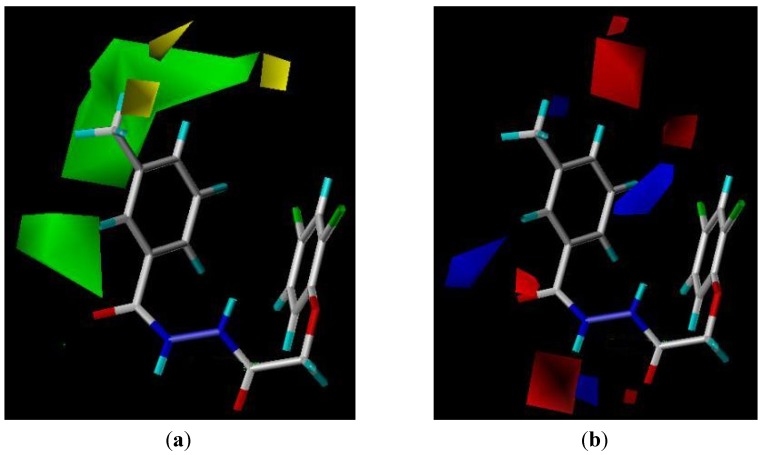
Steric and electrostatic contribution contour maps of CoMFA.

The CoMFA contour models are very similar, suggesting that for this training set, using four components is acceptable. Experimental and activities predicted by CoMFA for all compounds are listed in Table 4. As shown, a predictive CoMFA model was established with the conventional correlation coefficient r^2^ = 0.886 and the cross-validation coefficient q^2^ = 0.57. The plots of the predicted *versus* the actual activity values for all the compounds are shown in Figure 3. It is shown in Figure 4 that the contributions of steric and electrostatic fields (“stdev × coeff”) are 66.9% and 33.1% respectively.

With the CoMFA analysis, we obtained the isocontour diagrams of the steric and electrostatic field contributions (“stdev × coeff”), which is displayed in Figure 4. In Figure 4a, the steric field contours are represented with different colors: the green color at 3-position means a bulky group here would be favorable for higher herbicidal activity, while the yellow color means oppositely. As shown in Figure 4a, there is a green region located around the 3-position of the benzene ring, indicating that the bulky groups at this position will increase the herbicidal activity. This is in agreement with the actual experimental data: for example, compounds **4f** and **4g** have higher herbicidal activity with a bulky group in this position. In the same Figure 4b, the electrostatic contours are displayed in distinguishable colors: blue means an increase in the positive charge will lead to an increase in the activity, while the red contour defines in the opposite. So, the target compounds bearing an electron-withdrawing group at the 4-position of the benzene ring and an electron-donating group at the other positions displayed higher activity. These results provided useful information for further optimization of the compounds.

## 3. Experimental

### 3.1. Instruments

Melting points were determined using an X-4 apparatus (Beijing Tech Instruments Co., Beijing, China) and are uncorrected. ^1^H-NMR spectra were measured on a Bruker AC-P500 instrument (300 MHz, Bruker, Fallanden, Switzerland) using TMS as an internal standard and DMSO-*d*_6_ as solvent. Mass spectra were recorded on a Thermo Finnigan LCQ Advantage LC/mass detector instrument (Thermo Finnigan, MA, USA). Elemental analyses were performed on a Vario EL elemental analyzer. An LWMC-250 domestic microwave oven (Jiang-Ling Company, Nanjing, China) was used to do microwave reactions. All reagents are commercially available analytical grade or were synthesized by us.

### 3.2. General Procedure

2,4-Dichlorophenol (5 mmol), KI (1 mmol), DMF (1 mL), ethyl 2-chloroacetate (5 mmol) and TBAB (0.5 mmol) were place in a dried round–bottomed flask and the mixture was irradiated by microwaves (200 W) for 5 min. On completion of the reaction, the mixture was cooled to room temperature and then added to ethanol (10 mL) with constant stirring. After filtering off the inorganic salts, the reaction mixture was added to 85% hydrazine hydrate (5 mmol) and subjected to microwave irradiation (500 W) for an additional 2 min. Then, it was cooled to room temperature, allowed to settle for 1 h, and the precipitates were filtered off and recrystallized from ethanol to afford the pure product **3**. Then **3** (1 mmol) and substituted acyl chloride (1 mmol) were mixed in THF. The mixture was put into the microwave oven (400 W) and irradiated for 10 min to produce the crude solid, which on recrystallization with ethanol gave the pure product as shown in Scheme 1.

*N'-(2-(2,4-Dichlorophenoxy)acetyl)cyclopropanecarbohydrazide* (**4a**): White solid, yield 79%, mp 199–200 °C; ^1^H-NMR (DMSO-*d*_6_) δ: 0.58–0.78 (m, 4H, cyclopropane), 1.55–1.67 (m, 1H, cyclopropane), 4.70 (s, 2H, CH_2_O), 7.06 (d, *J* = 8.8 Hz, 1H, Ph), 7.35 (d, *J* = 8.9 Hz, 1H, Ph), 7.57 (s, 1H, Ph), 10.11 (s, 2H, NH); ESI-MS: 302.55 [M−H]^−^; Elemental analysis for C_12_H_12_Cl_2_N_2_O_3_: found C 47.45, H 4.08, N 9.31; calcd. C 47.54, H 3.99, N 9.24.

*N'-(2-(2,4-Dichlorophenoxy)acetyl)benzohydrazide* (**4b**): White solid, yield 90%, mp 148–150 °C; ^1^H-NMR (DMSO-*d*_6_) δ: 4.80 (s, 2H, CH_2_O), 7.35–7.59 (m, 5H, Ph), 7.84–7.92 (m, 3H, Ph), 10.22 (s, 1H, NH), 10.47 (s, 1H, NH); ESI-MS: 338.54 [M−H]^−^; Elemental analysis for C_15_H_12_Cl_2_N_2_O_3_: found C 52.95, H 3.46, N 8.54; calcd. C 53.12, H 3.57, N 8.26.

*N'-(2-(2,4-Dichlorophenoxy)acetyl)-4-nitrobenzohydrazide* (**4c**): White solid, yield 91%, mp 209–211 °C; ^1^H-NMR (DMSO-*d*_6_) δ: 4.82 (s, 2H, CH_2_O), 7.13 (d, *J* = 9.9 Hz, 1H, Ph), 7.36–7.40 (m, 1H, Ph), 7.59 (s, 1H, Ph), 8.00 (d, *J* = 8.3 Hz, 1H, Ph), 8.34 (d, *J* = 8.3 Hz, 1H, Ph), 10.39 (s, 1H, NH), 10.85 (s, 1H, NH); ESI-MS: 383.13 [M−H]^−^; Elemental analysis for C_15_H_11_Cl_2_N_3_O_5_: found C 47.02, H 3.11, N 11.12; calcd. C 46.90, H 2.89, N 10.94.

*4-Chloro-N'-(2-(2,4-dichlorophenoxy)acetyl)benzohydrazide* (**4d**): White solid, yield 88%, mp 199–201 °C; ^1^H-NMR (DMSO-*d*_6_) δ: 4.79 (s, 2H, CH_2_O), 7.14 (d, *J* = 8.9 Hz, 1H, Ph), 7.38 (d, *J* = 8.9 Hz, 1H, Ph), 7.55–7.61 (s, 3H, Ph), 7.86 (d, *J* = 8.6 Hz, 1H, Ph), 10.27 (s, 1H, NH), 10.57 (s, 1H, NH); ESI-MS: 371.93 [M−H]^−^; Elemental analysis for C_15_H_11_Cl_3_N_2_O_3_: found C 48.12, H 3.11, N 7.88; calcd. C 48.22, H 2.97, N 7.50.

*N'-(2-(2,4-Dichlorophenoxy)acetyl)-4-fluorobenzohydrazide* (**4e**): White solid, yield 85%, mp 173–175 °C; ^1^H-NMR (DMSO-*d*_6_) δ: 4.79 (s, 2H, CH_2_O), 7.14 (d, *J* = 8.9 Hz, 1H, Ph), 7.29–7.39 (m, 3H, Ph), 7.59 (d, *J* = 6.5 Hz, 1H, Ph), 7.90–7.94 (m, 2H, Ph), 10.39 (s, 2H, NH); ESI-MS: 356.12 [M−H]^−^; Elemental analysis for C_15_H_11_Cl_2_FN_2_O_3_: found C 50.23, H 3.08, N 8.02; calcd. C 50.44, H 3.10, N 7.84.

*N'-(2-(2,4-Dichlorophenoxy)acetyl)-3-methylbenzohydrazide* (**4f**): White solid, yield 82%, mp 160–162 °C; ^1^H-NMR (DMSO-*d*_6_) δ: 2.33 (s, 3H, CH_3_), 4.75 (s, 2H, CH_2_O), 7.14 (d, *J* = 8.9 Hz, 1H, Ph), 7.32–7.37 (m, 3H, Ph), 7.32–7.37 (m, 3H, Ph), 7.54–7.62 (m, 2H, ph), 7.66 (s, 1H, Ph), 10.34 (s, 2H, NH) ; ESI-MS: 352.65 [M−H]^−^; Elemental analysis for C_16_H_14_Cl_2_N_2_O_3_: found C 54.78, H 4.22, N 8.00; calcd. C 54.41, H 4.00, N 7.93.

*3-Chloro-N'-(2-(2,4-dichlorophenoxy)acetyl)benzohydrazide* (**4g**): White solid, yield 84%, mp 170–172 °C; ^1^H-NMR (DMSO-*d*_6_) δ: 4.76 (s, 2H, CH_2_O), 7.11 (d, *J* = 8.9 Hz, 1H, Ph), 7. 37 (d, *J* = 8.9 Hz, 1H, Ph), 7.40–7.59 (m, 3H, ph), 7.80 (d, *J* = 8.9 Hz, 1H, Ph), 7.87 (s, 1H, Ph), 10.46 (s, 2H, NH); ESI-MS: 371.64 [M−H]^−^; Elemental analysis for C_15_H_11_Cl_3_N_2_O_3_: found C 48.45, H 2.78, N 7.33; calcd. C 48.22, H 2.97, N 7.50.

*N'-(2-(2,4-Dichlorophenoxy)acetyl)-2-fluorobenzohydrazide* (**4h**): White solid, yield 81%, mp 148–150 °C; ^1^H-NMR (DMSO-*d*_6_) δ: 4.79 (s, 2H, CH_2_O), 7.12 (d, *J* = 8.9 Hz, 1H, Ph), 7.27–7.38 (m, 3H, Ph), 7.53–7.61 (m, 3H, ph), 10.33 (s, 2H, NH); ESI-MS: 356.44 [M−H]^−^; Elemental analysis for C_15_H_11_Cl_2_FN_2_O_3_: found C 50.56, H 3.33, N 8.02; calcd. C 50.44, H 3.10, N 7.84.

*2-Chloro-N'-(2-(2,4-dichlorophenoxy)acetyl)benzohydrazide* (**4i**): White solid, yield 90%, mp 186–188 °C; ^1^H-NMR (DMSO-*d*_6_) δ: 4.79 (s, 2H, CH_2_O), 7.13 (d, *J* = 8.9 Hz, 1H, Ph), 7. 36 (d, *J* = 8.9 Hz, 1H, Ph), 7.41–7.50 (m, 4H, ph), 7.59 (d, *J* = 2.6 Hz, 1H, Ph), 10.40 (s, 2H, NH); ESI-MS: 371.23 [M−H]^−^; Elemental analysis for C_15_H_11_Cl_3_N_2_O_3_: found C 48.44, H 3.12, N 7.78; calcd. C 48.22, H 2.97, N 7.50.

*2,4-Dichloro-N'-(2-(2,4-dichlorophenoxy)acetyl)benzohydrazide* (**4j**): White solid, yield 91%, mp 169–171 °C; ^1^H-NMR (DMSO-*d*_6_) δ: 4.77 (s, 2H, CH_2_O), 7.13 (d, *J* = 6.8 Hz, 1H, Ph), 7.34–7.39 (dd, *J* = 2.6 Hz, *J* = 2.6 Hz, 1H, Ph), 7.43–7.54 (m, 2H, Ph), 7.59 (d, *J* = 2.6 Hz, 1H, Ph), 7.71 (s, 1H, Ph), 10.48 (bs, 2H, NH); ESI-MS: 405.88 [M−H]^−^; Elemental analysis for C_15_H_10_Cl_4_N_2_O_3_: found C 44.33, H 2.44, N 7.09; calcd. C 44.15, H 2.47, N 6.86.

*N'-(2-(2,4-Dichlorophenoxy)acetyl)-2-methoxybenzohydrazide* (**4k)**: White solid, yield 99%, mp 175–177 °C; ^1^H-NMR (DMSO-*d*_6_) δ: 3.86 (s, 3H, OCH_3_), 4.76 (s, 2H, CH_2_O), 7.03 (t, *J* = 7.4 Hz, 1H, Ph), 7.14 (d, *J* = 7.8 Hz, 2H, Ph), 7.32–7.37 (m, 1H, Ph), 7.47–7.51 (m, 1H, Ph), 7.55–7.58 (m, 1H, Ph), 7.71 (d, *J* = 7.6 Hz, 1H, Ph), 10.26 (bs, 2H, NH); ESI-MS: 368.13 [M−H]^−^; Elemental analysis for C_16_H_14_Cl_2_N_2_O_4_: found C 51.95, H 4.08, N 7.89; calcd. C 52.05, H 3.82, N 7.59.

*N'-(2-(2,4-Dichlorophenoxy)acetyl)-4-methoxybenzohydrazide* (**4l**): White solid, yield 79%, mp 174–175 °C; ^1^H-NMR (DMSO-*d*_6_) δ: 3.80 (s, 3H, OCH_3_), 4.78 (s, 2H, CH_2_O), 7.00 (d, *J* = 8.8 Hz, 2H, Ph), 7.14 (d, *J* = 8.9 Hz, 1H, Ph), 7.35 (d, *J* = 8.9 Hz, 1H, Ph), 7.58 (s, 1H, Ph), 7.83 (d, *J* = 8.8 Hz, 2H, Ph), 10.26 (s, 2H, NH); ESI-MS: 368.45 [M−H]^−^; Elemental analysis for C_16_H_14_Cl_2_N_2_O_4_: found C 52.11, H 4.02, N 7.87; calcd. C 52.05, H 3.82, N 7.59.

*N'-(2-(2,4-Dichlorophenoxy)acetyl)-4-iodobenzohydrazide* (**4m**): White solid, yield 88%, mp 230–231 °C; ^1^H-NMR (DMSO-*d*_6_) δ: 4.79 (s, 2H, CH_2_O), 7.12 (d, *J* = 8.6 Hz, 1H, Ph), 7.35–7.39 (m, 1H, Ph), 7.59 (d, *J* = 8.9 Hz, 2H, Ph), 7.63 (s, 1H, Ph), 7.87 (d, *J* = 8.9 Hz, 2H, Ph), 10.26 (s, 1H, NH), 10.56 (s, 1H, NH); ESI-MS: 463.88 [M−H]^−^; Elemental analysis for C_15_H_11_Cl_2_IN_2_O_3_: found C 38.98, H 2.54, N 6.23; calcd. C 38.74, H 2.38, N 6.02.

*N'*-*(2-(2,4-Dichlorophenoxy)acetyl)-5-methylisoxazole-4-carbohydrazide* (**4n**): White solid, yield 99%, mp 118–120 °C; ^1^H-NMR (DMSO-*d*_6_) δ: 2.62 (s, 3H, Het-CH_3_), 4.79 (s, 2H, CH_2_O), 7.10 (d, *J* = 8.9 Hz, 1H, Ph), 7.37 (d, *J* = 8.9 Hz, 1H, Ph), 7.58 (s, 1H, Ph), 8.90 (s, 1H, Het-CH), 10.27 (s, 1H, NH), 10.37 (s, 1H, NH); ESI-MS: 343.15 [M−H]^−^; Elemental analysis for C_13_H_11_Cl_2_N_3_O_4_: found C 45.66, H 3.56, N 12.31; calcd. C 45.37, H 3.22, N 12.21.

*1-Cyano-N'-(2-(2,4-dichlorophenoxy)acetyl)cyclopropanecarbohydrazide* (**4o**): White solid, yield 98%, mp 188–190 °C; ^1^H-NMR (DMSO-*d*_6_) δ: 1.51–1.64 (m, 4H, cyclopropane), 4.75 (s, 2H, CH_2_O), 7.00 (d, *J* = 6.0 Hz, 1H, Ph), 7.33 (d, *J* = 6.1 Hz, 1H, Ph), 7.57 (s, 1H, Ph), 10.31 (s, 2H, NH); ESI-MS: 327.66 [M−H]^−^; Elemental analysis for C_13_H_11_Cl_2_N_3_O_3_: found C 47.45, H 3.43, N 12.98; calcd. C 47.58, H 3.38, N 12.81.

*N'-(2-(2,4-Dichlorophenoxy)acetyl) butyrohydrazide* (**4p**): White solid, yield 96%, mp 162–164 °C; ^1^H-NMR (DMSO-*d*_6_) δ: 0.84 (t, *J* = 7.3 Hz, 3H, CH_3_), 1.54 (q, *J* = 7.3 Hz, 2H, CH_2_), 2.00 (t, *J* = 7.2 Hz, 2H, CH_2_), 4.57 (s, 2H, CH_2_O), 7.05 (d, *J* = 8.9 Hz, 1H, Ph), 7.34 (d, *J* = 8.9 Hz, 1H, Ph), 7.56 (s, 1H, Ph), 9.23 (s, 1H, NH), 9.95 (s, 1H, NH); ESI-MS: 304.12 [M−H]^−^; Elemental analysis for C_12_H_14_Cl_2_N_2_O_3_: found C 47.44, H 4.78, N 9.23; calcd. C 47.23, H 4.62, N 9.18.

*N'-(2-(2,4-Dichlorophenoxy)acetyl)isobutyrohydrazide* (**4q**): White solid, yield 92%, mp 174–176 °C; ^1^H-NMR (DMSO-*d*_6_) δ: 0.82 (d, *J* = 6.5 Hz, 6H, CH_3_), 1.99–2.11 (m, 1H, CH), 4.70 (s, 2H, CH_2_O), 7.01 (d, *J* = 9.0 Hz, 1H, Ph), 7.33 (d, *J* = 6.6 Hz, 1H, Ph), 7.56 (s, 1H, Ph), 9.97 (s, 2H, NH); ESI-MS: 304.95 [M−H]^−^; Elemental analysis for C_12_H_14_Cl_2_N_2_O_3_: found C 47.11, H 4.44, N 10.36; calcd. C 47.23; H 4.62; N 9.18.

*2-(2,4-Dichlorophenoxy)-N'-(2-(2,4-dichlorophenoxy)acetyl)acetohydrazide* (**4r**): White solid, yield 92%, mp 214–216 °C; ^1^H-NMR (DMSO-*d*_6_) δ: 4.73 (s, 4H, CH_2_O), 7.01 (d, *J* = 9.0 Hz, 2H, Ph), 7.35 (d, *J* = 8.8 Hz, 2H, Ph), 7.58 (s, 2H, Ph), 10.26 (s, 2H, NH); ESI-MS: 437.95 [M−H]^−^; Elemental analysis for C_16_H_12_Cl_4_N_2_O_4_: found C 44.11, H 3.08, N 6.66; calcd. C 43.87, H 2.76, N 6.39.

*2-(2,4-Dichlorophenoxy)-N'-(2-(2,4-dichlorophenoxy)acetyl)propanehydrazide* (**4s**): White solid, yield 96%, mp 245–246 °C; ^1^H-NMR (DMSO-*d*_6_) δ: 1.46 (d, *J* = 6.5 Hz, 6H, CH_3_), 4.80 (q, *J* = 6.5 Hz, 2H, Me-CH-OAr), 7.05 (d, *J* = 8.8 Hz, 2H, Ph), 7.29–7.32 (d, *J* = 8.9 Hz, 2H, Ph), 7.55 (s, 2H, Ph), 10.30 (bs, 2H, NH); ESI-MS: 465 [M−H]^−^; Elemental analysis for C_18_H_16_Cl_4_N_2_O_4_: found C 46.12, H 3.23, N 6.23; calcd. C 46.38, H 3.46, N 6.01.

*N'-(2-(2,4-Dichlorophenoxy)acetyl)furan-3-carbohydrazide* (**4t**): White solid, yield 92%, mp 128–130 °C; ^1^H-NMR (DMSO-*d*_6_) δ: 4.72 (s, 2H, CH_2_O), 6.61 (s, 1H, Furan), 7.11–7.14 (m, 2H, Ph), 7.35 (m, *J* = 8.8 Hz, 1H, Furan), 7.57 (s, 1H, Furan), 7.82 (s, 1H, Ph), 10.31 (s, 2H, NH); ESI-MS: 328.00 [M−H]^−^; Elemental analysis for C_13_H_10_Cl_2_N_2_O_4_: found C 44.54, H 3.29, N 8.24; calcd. C 44.74, H 3.06, N 8.51.

*(2E,4Z)-N'-(2-(2,4-Dichlorophenoxy)acetyl)hexa-2,4-dienehydrazide* (**4u**): White solid, yield 98%, mp 132–134 °C; ^1^H-NMR (DMSO-*d*_6_) δ: 1.79 (d, *J* = 6.2 Hz, 3H, CH_3_), 4.73 (s, 2H, CH_2_O), 5.91 (d, *J* = 15.1 Hz, 1H, CH), 6.11–6.28 (m, 2H, CH), 7.03–7.14 (m, 2H, Ph), 7.35 (d, *J* = 8.9 Hz, 1H, Ph), 7.57 (s, 1H, Ph), 10.24 (s, 2H, NH); ESI-MS: 328.15 [M−H]^−^; Elemental analysis for C_14_H_14_Cl_2_N_2_O_3_: found C 50.95, H 4.44, N 8.88; calcd. C 51.08, H 4.29, N 8.51.

*N'-(2-(2,4-Dichlorophenoxy)acetyl)nicotinohydrazide* (**4v**): White solid, yield 88%, mp 197–199 °C; ^1^H-NMR (DMSO-*d*_6_) δ: 4.81 (s, 2H, CH_2_O), 7.14 (d, *J* = 8.9 Hz, 1H, Ph), 7.51–7.54 (m, 1H, Py), 7.58 (s, 1H, Ph), 8.19 (d, *J* = 8.0 Hz, 1H, Py), 8.74 (d, *J* = 3.2 Hz, 1H, Py), 9.00 (s, 1H, Py), 10.32 (s, 1H, NH), 10.69 (s, 1H, NH); ESI-MS: 339.56 [M−H]^−^; Elemental analysis for C_14_H_11_Cl_2_N_3_O_3_: found C 49.65, H 3.43, N 12.31; calcd. C 49.43, H 3.26, N 12.35.

*N'-(2-(2,4-Dichlorophenoxy)acetyl)isonicotinohydrazide* (**4w**): White solid, yield 92%, mp 103–105 °C; ^1^H-NMR (DMSO-*d*_6_) δ: 4.80 (s, 2H, CH_2_O), 7.15 (d, *J* = 8.9 Hz, 1H, Ph), 7.38 (d, *J* = 8.9 Hz, 1H, Ph), 7.52 (s, 1H, Ph), 7.74 (d, *J* = 5.9 Hz, 2H, Py), 8.75 (d, *J* = 5.9 Hz, 2H, Py), 10.35 (s, 1H, NH), 10.78 (s, 1H, NH); ESI-MS: 339.15 [M−H]^−^; Elemental analysis for C_14_H_11_Cl_2_N_3_O_3_: found C 49.19, H 3.32, N 12.53; calcd. C 49.43, H 3.26, N 12.35.

*2-(2,4-Dichlorophenoxy)-N'-(2-(2,4-dichlorophenoxy)acetyl)acetohydrazide* (**4x**): White solid, yield 92%, mp 214–216 °C; ^1^H-NMR (DMSO-*d*_6_) δ: 4.73 (s, 4H, CH_2_O), 7.01 (d, *J* = 9.0 Hz, 2H, Ph), 7.35 (d, *J* = 8.8 Hz, 2H, Ph), 7.58 (s, 2H, Ph), 10.26 (s, 2H, NH); ESI-MS: 437.95 [M−H]^−^; Elemental analysis for C_16_H_12_Cl_4_N_2_O_4_: found C 44.11, H 3.08, N 6.66; calcd. C 43.87, H 2.76, N 6.39.

### 3.3. 3D-QSAR Analysis

**Figure 5:**
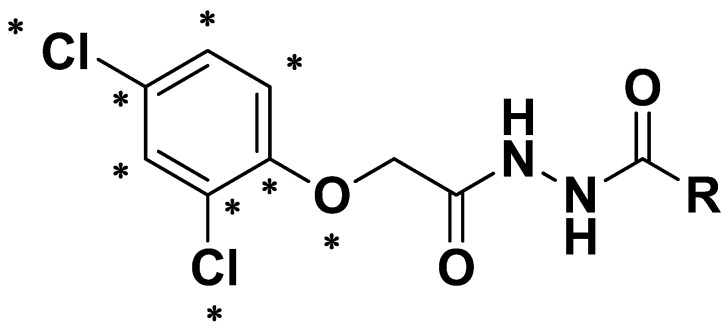
The asterisk skeleton of title compounds.

**Figure 6:**
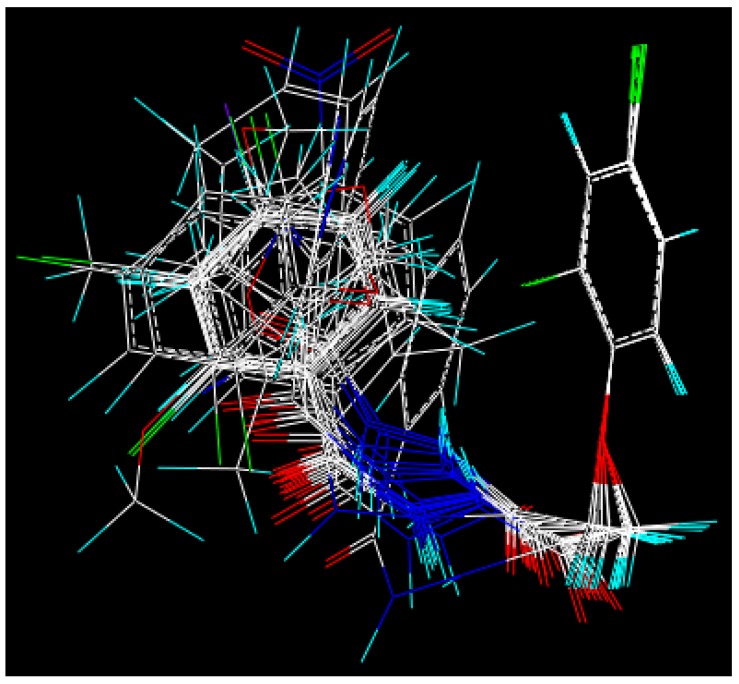
Superposition modes of compounds.

Molecular modeling was performed using SYBYL 6.91 software [27] (Tripos, Inc., St. Louis, MO, USA), and the CoMFA method was done according to our previous work [28]. The herbicidal activities of **24** compounds against *Digitaria sanguinalis* data (% I) at pre-emergence condition used to derive the CoMFA analyses model are listed in Table 5. The activity was expressed in terms of *p*IC_50_ by the formula *p*IC_50_ = log(I/((100 − I) × Mw)), where I is the percent inhibition and Mw is the molecular weight of the tested compounds. The compound **4f** was used as a template to build the other molecular structures. Because these compounds share a common skeleton, 16 atoms marked with an asterisk were used for rms-fitting onto the corresponding atoms of the template structure (Figure 5 and Figure 6).

**Table 5:** The structures, activities and total score of compounds.

No.	R	*p*IC_50_	*p*IC_50_'	Residue
**4a**	cyclopropyl	−2.06064	−2.1257	0.06506
**4b**	phenyl	−2.47998	−2.5328	0.05282
**4c**	*p*-nitrophenyl	−3.14439	−3.0982	−0.04619
**4d**	*p*-chlorophenyl	−2.50804	−2.5895	0.08146
**4e**	*p*-fluorophenyl	−0.55723	−0.6172	0.05997
4f *	*m*-methylphenyl	−3.4516	−3.5621	0.1105
**4g**	*m*-chlorophenyl	−3.27036	−3.5229	0.25254
**4h**	*o*-fluorophenyl	−2.784	−2.8013	0.0173
**4i**	*o*-chlorophenyl	−2.3074	−2.4085	0.1011
**4j** ^#^	2,4-dichlorophenyl	−2.61072	−2.8691	0.25838
**4k**	*o*-methoxyphenyl	−3.11189	−3.0097	−0.10219
**4l**	*p*-methoxyphenyl	−2.60028	−2.4112	−0.18908
**4m**	*p*-iodophenyl	−4.08294	−4.1954	0.11246
**4n**	5-methylisoxazole-4-yl	−2.73284	−2.6376	−0.09524
**4o**	1-CN-cyclopropyl	−4.51171	−4.2652	−0.24651
**4p** ^#^	propyl	−1.65895	−1.6622	0.00325
**4q**	isopropyl	−2.10825	−2.3056	0.19735
**4r**	2,4-dichlorophenoxymethyl	−3.09808	−3.2077	0.10962
**4s**	(2-(2,4-dichlorophenoxy)acetyl)propyl	−1.82968	−1.9661	0.13642
**4t**	furan	−2.28624	−2.1627	−0.12354
**4u**	(2*E*,4*Z*)- hexa-2,4-diene-	−2.85481	−2.6382	−0.21661
**4v** ^#^	3-pyridine	−2.9882	−2.7025	−0.2857
**4w**	4-pyridine	−2.23	−2.3669	0.1369

Note: *p*IC_50_ = Experimental value, *p*IC_50_' = predictive value of *p*IC_50_, * template molecule, **^#^** test.

Each structure was fully geometry-optimized using a conjugate gradient procedure based on the TRIPOS force field and Gasteiger and Hückel charges. Because these compounds share a common skeleton, 10 atoms marked with an asterisk were used for rms-fitting onto the corresponding atoms of the template structure. CoMFA steric and electrostatic interaction fields were calculated at each lattice intersection on a regularly spaced grid of 2.0 Å. The grid pattern was generated automatically by the SYBYL/CoMFA routine, and an *sp*^3^ carbon atom with a van der Waals radius of 1.52 Å and a +1.0 charge was used as the probe to calculate the steric (Lennard-Jones 6-12 potential) field energies and electrostatic (Coulombic potential) fields with a distance-dependent dielectric at each lattice point. Values of the steric and electrostatic fields were truncated at 30.0 kcal/mol. The CoMFA steric and electrostatic fields generated were scaled by the CoMFA-STD method in SYBYL. The electrostatic fields were ignored at the lattice points with maximal steric interactions. A partial least-squares (PLS) approach was used to derive the 3D-QSAR, in which the CoMFA descriptors were used as independent variables, and ED values were used as dependent variables. The cross-validation with the leave-one-out (LOO) option and the SAMPLS program, rather than column filtering, was carried out to obtain the optimal number of components to be used in the final analysis. After the optimal number of components was determined, a non-cross-validated analysis was performed without column filtering. The modeling capability (goodness of fit) was judged by the correlation coefficient squared, r^2^, and the prediction capability (goodness of prediction) was indicated by the cross-validated r^2^ (q^2^).

### 3.4. Herbicidal Activities Assay

*Plant Material*. Two dicotyledonous weeds, rape (*Brassica napus*) and amaranth pigweed (*Amaranthus retroflexus*), and two monocotyledonous crops, barnyardgrass (*Echinochloa crus-galli*) and hairy crabgrass (*Digitaria sanguinalis*), were used to test the herbicidal activities of the title compounds. The seeds of amaranth pigweed were reproduced outdoors and stored at room temperature. 2,4-dichlorophenoxyl acetic acid (2,4-D) was obtained as a commercial material.

*Culture Method*. The seeds were planted in 6 cm diameter plastic boxes containing artificial mixed soil. Before plant emergence, the boxes were covered with plastic film to retain moisture. Plants were grown in the green house. The fresh weight of the above ground tissues was measured 17 days after treatment. The inhibition percent was used to describe the control efficiency of the compounds.

*Treatment*. The dosage (activity ingredient) for each compound is corresponded to 1500 g/ha. The purified compounds were dissolved in 100 μL of *N*,*N*-dimethylformamide with 0.1 μL of Tween 20, and then prepared for spraying with a laboratory belt sprayer delivering a 750 L/ha spray volume. Compounds and 2,4-D were sprayed immediately after seed planting (preemergence treatment) or after the expansion of the first true leaf (postemergence treatment). The mixture of same amount of water, *N*,*N*-dimethylformamide, and Tween 20 was sprayed as the blank. Each treatment was triplicated. The activity numbers represented the percent displaying herbicidal damage as compared to the blank. The error of the experiments was 2%.

### 3.5. Plant Growth Regulatory Activity Assay

After dipping into distilled water for 1 h at 23 °C, the cucumber seeds (Jinke, no. 4, commercially available) were then sown into the soil with 0.7% agar on a covered porcelain enamel plate and incubated at 26 °C in a darkroom for 3 days. The same sized cotyledons were carefully selected for the subsequent biological assay. The analyte (3 mg) was resolved in *N*,*N*-dimethyl formamide (3 mL) and this solution was then diluted to 10% concentration with distilled water. A sample solution (0.3 mL) was sprayed over a 6 cm diametered filter paper and solvent was volatilized to dryness on air. The filter paper thus prepared was placed into a 6 cm diameter incubation vessel and soaked with 10 cm distilled water. Finally, 10 pieces of cotyledon of the same size were added into the incubation vessel. These cotyledons were incubated at 26 °C in a darkroom for 5 days. Then the rhizogenesis numbers of every 10 pieces of hypocotyls were measured. Each sample was repeated twice. In contrast, the distilled water was used as a blank experiment. The relative ratios of cucumber cotyledon rhizogenesis were calculated according to the following formula:

Relative ratio % = (*N*_S_ − *N*_C_)/*N*_C_ × 100%

where *N*_S_ and *N*_C_ are the numbers of cucumber cotyledon rhizogenesis of tested compound and control experiment, respectively.

## 4. Conclusions

In summary, a series of diacylhydrazine derivatives containing 2,4-dichlorophenoxy moieties were synthesized in good yields. The preliminary bioassays showed that some of the compounds had good herbicidal activity. Structure–activity relationship and comparative molecular field analysis (CoMFA) studies was done. The present findings provided a powerful complement to the SARs of herbicides, and warrant future investigation of the mechanism of action of these new analogues.
